# A cross-sectional study on exploring the antecedents of patient’s revisit intention: Mediating role of trust in the hospital among patients in India

**DOI:** 10.12688/f1000research.128220.1

**Published:** 2023-01-19

**Authors:** Nahima Akthar, Smitha Nayak, Yogesh Pai P

**Affiliations:** 1 Ph.D. Scholar, Manipal Institute of Management, Manipal Academy of Higher Education, Manipal, India; 2 Associate Professor, Department of Humanities and Management, Manipal Institute of Technology, Manipal Academy of Higehr Education, Manipal, India; 3 Associate Professor - Senior Scale and Head of the Department, Department of Humanities and Management, Manipal Institute of Technology, Manipal Academy of Higher Education, Manipal, India

**Keywords:** Healthcare, service quality, satisfaction, trust, revisit intention, structural equation modeling, adverse services

## Abstract

Background: In the healthcare domain, patients’ trust in the hospital plays an instrumental role in determining the behavioral intention of the patient. This article attempts to investigate the impact of service quality perception on behavioral intention with the mediating effect of trust in the hospital and patient satisfaction.

Methods: This research was carried out in multispecialty hospitals located in Bangalore Urban and Mysore districts of Karnataka during August 2021. This was a questionnaire-based study and the sample size was 242. Statistical Package for the Social Science (SPSS) 27.0 and SmartPLS 3.0 software was used to analyze the data.

Results: The findings revealed that perceived service quality significantly influences trust through patient satisfaction (observed partial mediation) and patient satisfaction significantly impacts behavioral intention through trust (observed partial mediation).

Conclusion: This study empowers hospital managers to understand the factors influencing behavioral intention. Healthcare professionals must ensure that good quality service is delivered to enhance patient satisfaction and trust in adverse services, which influence behavioral intention among the patients.

## Introduction

The service sector has a substantial influence on a country’s development and is increasingly driving economic transformation. The service sector accounted for approximately 55% of the gross domestic product (GDP) and 45% of employment in developing economies in 2019. ^1^ The service sector constitutes diverse services like healthcare, banking, hospitality, consultancy, and entertainment, to name a few. In services like healthcare, customer undergoes stressful situation, in terms of treatment process etc., hence create an unpleasant experience for the customer. ^2^^,^^3^ Healthcare service providers are leaving no stone unturned to create a positive servicescape to nurture an enabling environment for patients. Hence, service quality has attracted significant research and academic interest. Patient expectations for increasing levels of healthcare service quality are primarily triggered by rising healthcare demand and informed consumer decision-making. ^4^^–^^6^

Researchers have shown that healthcare services are evaluated based on service quality and medical efficacy. ^7^^–^^10^ Service quality (SQ) is termed as the process of meeting consumers’ expectations by providing excellent services. ^11^ Service quality evaluation helps to identify the unmet needs of the patients during the service encounter. In the healthcare landscape, SQ is crucial and it has a meaningful effect on patient satisfaction (PS). ^12^^–^^16^

Patients evaluate healthcare service quality based on their personal experiences, therefore a user-centered approach to healthcare delivery is critical for increasing overall patient satisfaction. ^4^^,^^17^ Patients believe that the Health Care Organization (HCO) meets their expectations when they receive essential healthcare services, which results in a strong bond between the patients and the HCO. ^18^ Patients’ perceptions of medical staff’s trustworthiness and the degree of satisfaction with the care they receive are directly related to each other. As a result, when patients require healthcare services again, they will seek the same facility and physician. Researchers have discovered that SQ is a precursor to PS, and satisfaction is an antecedent to trust. ^12^^,^^19^

When patients receive satisfactory medical care, they trust the hospital and return for future medical treatment. ^20^^–^^22^ Patients shift to another hospital if the SQ of the hospital declines. ^4^ Patients’ willingness to visit the hospital again for their treatment is referred to as “intent to revisit”. ^23^ The effect of hospital SQ on PS and intent to return has been investigated by the researchers. ^24^^,^^25^ Patients’ revisit intent and the services rendered by healthcare professionals are also studied in the past. ^16^ This is in line with the service marketing literature, which reiterates the importance of trust in the service provider and trust in the brand of the service provider. ^26^ The “Commitment-Trust Theory (CTT)” proposed by Morgan and Hunt (1994) has shown the relevance of trust and commitment in developing effective long-term partnerships in the service industry. ^27^^,^^28^ Relationships are established on the foundation of mutual commitment in the services relationship marketing area. ^29^ Customer commitment is defined as the customer’s long-term aim to create and maintain a connection with the supplier. ^30^ Furthermore, commitment is a feature that has been widely identified in earlier studies as a key predictor of sustained usage. In addition, it was found that dedication had a considerable beneficial impact on consumers’ recommendations. Loyal consumers assume the role of company advocates by spreading favorable word of mouth and recommending the service to others. ^31^

In the Jordanian setting, researchers have explored the association between perceived service quality (PSQ), PS, and trust in the service provider. ^12^ They opined that trust in the service provider played an instrumental role in determining perceived service quality and patient satisfaction. The association of patients’ revisit intention has been researched with constructs like satisfaction, medical SQ, hospital brand image, assurance, and word-of-mouth. ^24^^,^^25^^,^^32^ Researchers have explored the constructs such as PSQ, customer satisfaction, perceived value, brand trust, loyalty, and behavioral intention (BI) in various service sectors. ^33^^–^^36^ However, literature on influencing BI through PSQ, PS, and trust in the hospital in the Indian context has received less attention. Therefore, this study proposes a conceptual framework and tests the relationship between the constructs in an attempt to bridge the gap. The objectives of the study are:

• To investigate the mediating effect of patient satisfaction between perceived service quality and trust in the hospital
• To examine the role of trust in mediating the relationship between patient satisfaction and behavioral intention

This research paper is structured as follows: First, a review of the literature and hypothesis development is explained. Secondly, the methodology adopted and results are presented. The report concludes with a discussion of the results, limitations, and useful implications, as well as recommendations for additional research.

## Review of Literature

### Perceived service quality (PSQ)

Service quality (SQ) is regarded as a vital aspect of modern service firms. ^37^ “SQ is the delivery of excellent or superior service relative to customer expectations”. ^11^ “PSQ is defined as the consumer’s judgment about a product’s overall excellence or superiority”. ^38^ In hospitals, SQ is appraised based on care provided by healthcare personnel, not only the technical aspect. ^6^ Researchers have proposed that healthcare SQ acts as a mirror of PS. ^13^ Also, SQ and consumer loyalty are strongly associated with each other. ^39^^,^^40^ In addition, SQ is a key element to an effective consumer relationship. ^18^^,^^41^

### Patient satisfaction (PS)

“Satisfaction is defined as the consumer’s fulfillment response, a post-consumption judgment by the consumer that a service provides a pleasing level of consumption-related fulfillment including under or over fulfillment”. ^42^ Like other services, customer or patient satisfaction is also considered to be a significant service outcome. PS is deliberated as one of the fundamental parameters of healthcare quality. Satisfaction among patients is a multi-dimensional concept. ^17^ “PS is a patient’s sensation of joy or disappointment as a result of comparing a perceived service performance to his or her expectations”. ^43^

PS is a result of a patient’s experience of receiving medical services, and it displays a variety of functions concerning consumer relationships. ^17^ Researchers have suggested that satisfaction is known as a global consumer response since it results from consumers’ pleasure levels. ^43^ Healthcare professionals must ensure that they provide good quality services to meet patients’ anticipations thereby improving satisfaction. Therefore, hospitals must work consistently to meet their patient’s expectations. ^12^^,^^44^

### Trust

“Trust is the client’s confidence that the service provider will fulfill his/her expectations by delivering what was promised explicitly and implicitly”. ^45^ An organization must maintain customers’ trust, which will enhance competition and accomplishment of goals. ^46^ A service provider is expected to work in favor of the patient when there is trust between them. ^47^ “Trust is defined as a willingness to rely on an exchange partner in whom one has confidence”. ^48^ Trust arises from faith and reliability. Hence, good relationships are developed and maintained. ^26^ Positive behavioral intents are developed as a result of mutual trust among the partners. Trust inspires both parties to maintain the relationships. ^16^^,^^49^ Through repeated encounters, trust is gradually created, and these interactions influence customers’ revisit intention. ^50^

“Patient trust is defined as patient’s belief that their doctors would act in their best interests and offer proper treatment and medical care”. ^51^ Researchers have suggested that trust is essential since it serves as a foundation for upcoming associations. Patients usually evaluate the services provided by the healthcare professionals which leads to trust in the hospital brand. ^30^^,^^52^ Customers revisit the service entity when they trust that service brand, which results in increasing the profit. ^52^ Researchers have observed that building trust in the healthcare brand is very essential for selecting a hospital for treatment and continuing the treatment with the same hospital. ^16^^,^^53^

### Behavioral Intention (BI)

“BI can be defined as the likelihood that an individual will take part in a particular behavior and represents the level of effort that an individual is willing to make to secure a specific behavior”. ^54^ BI concerns the hospital talks about the preparedness of patients to come back to the same hospital for their treatment. Retaining a client is very essential for the long-standing achievement of any service provider. ^25^ Researchers have proposed that if a service is evaluated positively, then the desired BI of the client build up liaison with the provider. BI is divided into desirable and undesirable intentions. Clients with desirable BI think optimistically about the provider and recommend it to others. ^55^

The key elements of BI are “SQ”, “customer satisfaction”, “perceived value”, and “customer loyalty”. ^56^ Past research shows that client behavior is critical to the profitability and long-term viability of any service business. ^57^ Customer satisfaction results from service contact having a direct impact on consumer behavior. Satisfied customers are more likely to experience positive behavior and stick to the same service provider. Client pleasure not only increases the company’s relationship with its customers but also aids in customer retention. ^58^^,^^59^

### Perceived service quality and patient satisfaction

“Satisfaction is defined as an assessment of service experience, either specific or global, influenced by SQ among other factors”. ^60^ The relationship between SQ and customer satisfaction (CS) is well-established by researchers from various sectors. ^24^^,^^61^^,^^62^ Researchers view SQ as a precursor of CS. ^37^^,^^62^^,^^63^ In HCO, the relationship between healthcare quality and PS is significant. ^25^^,^^43^^,^^63^ When patients’ necessities and anticipations are equal to the quality services of the hospital, the patient will be satisfied. ^64^^,^^65^ A linear liaison between SQ and PS (increased satisfaction level) is observed when the SQ is at a higher level. ^13^^,^^25^ Hence, it is expected that there is a significant association between PSQ and PS.

***HI:** PSQ is significantly related to PS.*

### Patient satisfaction and trust

Patient satisfaction serves as a key factor when evaluating the quality of healthcare services. ^7^^,^^25^^,^^44^ Based on an assessment of their impression and service outcome expectations, customers are either satisfied or dissatisfied. ^66^ PS is a multi-faceted aspect of the healthcare industry that has an impact on patient trust. ^12^^,^^43^ Trust is denoted as a key factor in any relationship. It is crucial since it is the most important part of any exchange connection. ^53^^,^^67^

According to past research, CS is critical for building customer trust and loyalty. ^45^^,^^68^ Researchers have witnessed that satisfaction is a precursor of trust. ^69^^,^^70^ Scholars have revealed that CS and trust play a crucial role in service sectors like tourism, ^45^ retail, ^71^ and public services. ^66^ Trust can be developed over some time, based on CS. ^19^^,^^72^ Customer trust is regarded as a crucial construct concerning satisfaction in marketing and consumer behavior research. ^21^ Therefore, we propose that PS has a significant positive influence on trust in the hospital.

***H2:** PS is positively related to trust in the hospital.*

### The mediating role of PS

PS results from healthcare SQ which leads to trust in the hospital. PS is a processing feature of trust. ^12^ Perceived quality has a large and beneficial effect on consumer trust. SQ is a key precursor to customer trust. ^28^^,^^72^ CS is a mediator in the relationship between SQ and consumer trust. ^12^^,^^19^ Hence, we tend to establish that the liaison between PSQ and trust in the hospital is mediated by PS.

***H3:** The link between PSQ and trust in the hospital is mediated by PS.*

### Trust and BI

The effect of trust on BI has been exhaustively studied in the marketing literature. ^30^^,^^32^^,^^49^^,^^73^ Customers’ BI can be developed and maintained with the use of trust. ^67^^,^^74^ Trust and commitment are the main drivers of intent to return. Customers’ intentions to recommend the brand and make repeat purchases are also influenced by their degree of trust in the company. ^23^

In service sectors, the association between brand trust and revisit intention is studied previously. ^75^^,^^76^ Maintaining customer-provider relationships requires a high level of trust. ^16^^,^^49^^,^^74^ Overall, past empirical research supports the idea that trust is important in the establishment of BI. The trust–BI relationship has received a lot of attention and support, especially in the context of the service sector. ^40^^,^^46^ Hence, we propose that trust in the hospital significantly contributes to BI of the customer.

***H4:** Trust in the hospital is positively related to BI.*

### The mediating role of trust

The first research on healthcare trust concentrated on the interpersonal trust of the patient in their doctors. ^51^ Scholars have investigated the impact of trust and assurance on “relationship marketing performance”. ^26^ Trust has been studied as a mediator in other business entities. ^50^^,^^77^^,^^78^ In a hospital, trust is important in the interaction between healthcare personnel and patients. ^16^^,^^79^^,^^80^

Scholars have observed that a positive service evaluation (cognition response) has a favorable impact on patients’ trust in healthcare interactions. ^81^ Trust has been established as a strong mediator among PSQ and intent to return to avail medical care. ^16^ Patients’ perceptions of medical staff’s trustworthiness have an impact on their satisfaction level through the care they received. Studies have found that customers develop trust in the organization only if they are satisfied with the service provider. ^21^^,^^67^ Hence, we tend to establish that the liaison between PS and BI is mediated by trust in the hospital.

***H5:** The association between PS and BI is mediated by trust in the hospital.*

**Figure 1.**
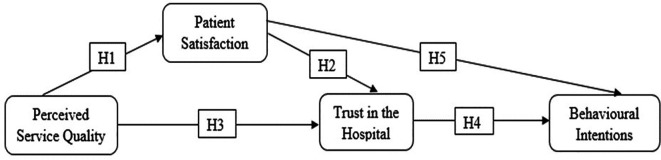
Proposed conceptual framework.

The proposed conceptual framework ( Figure 1) suggests that PSQ influences BI through PS and trust in the hospital.

## Methods

### Study design

This research endeavor is quantitative by nature and has adopted a “cross-sectional research design”. The research philosophy deliberated in this study is “post-positivism”, which deals with the development of empirical approaches to interpret and study the behavior of people. This study explores the association between the independent, dependent, and mediating variables as proposed in the conceptual framework.

### Study setting

There is significant research evidence to indicate a strong relationship between human development index (HDI) and life satisfaction. ^82^^,^^83^ Districts were ranked based on the HDI report of Karnataka state. ^84^: 4 districts ranked high as per the HDI report namely, Bangalore Urban, Dakshina Kannada, Udupi, and Mysore. For this study, Bangalore Urban and Mysore Districts were selected through a simple random sampling approach. This research was undertaken in three multispecialty hospitals located in Bangalore Urban and one located in Mysore during 4 ^th^ August 2021 to 26 ^th^ August 2021.

### Eligibility criteria

The study comprised of patients within the 18-65 years of age group, who knew English or Kannada, and consulted the outpatient department at least two times. Medicine and medical specializations, as well as surgery and surgical specialties, were the outpatient departments (OPDs) studied. Pediatric and psychiatric OPDs were not included in the study.

### Data Sources/Measurements

A structured questionnaire was used to gather the data. A copy of the questionnaire can be found in the *Extended data.* ^85^ The survey included scales for assessing SQ perceptions, ^86^^,^^87^ PS, ^42^^,^^88^ trust, ^47^ and BI. ^56^ All the scales were scored on a “five-point Likert scale”. The questionnaire featured a total of 27 questions, 6 of which were about the participants’ demographics and 21 of which were about the study’s constructs. The questionnaire was developed in English and translated into the Kannada language in Microsoft Word 2013. The printed version of the questionnaire was utilized for data collection. Participation in the study was voluntary. The respondents were given a participant information sheet that explained the study’s purpose and procedures. After obtaining written agreement from the subjects, the investigator handed over the questionnaire to them. The research subjects were directed by the researcher to mark the right point on the questionnaire. The questionnaire was completed by all 242 participants; no partially completed questionnaires were acquired. The data were collected during 4 ^th^ August 2021 to 26 ^th^ August 2021.

### Study size

This study adopted the purposive sampling method. A total of 242 respondents participated in this study. Respondents were contacted at the pharmacy, which was their last point of contact with the hospital for outpatient services.

The sample size was generated by multiplying the number of elements on the rating scale by ten ^89^ i.e. 21*10 = 210. Considering 10% of the unanswered sample (i.e.21) gave rise to 231 (210+21=231). Finally, the researcher approached 242 participants to collect the data.

### Statistical methods

The preliminary analysis was performed using IBM SPSS statistics 27 (IBM SPSS Statistics, RRID: SCR_016479; Armonk, NY: IBM Corp). The frequency distribution of the demographic variables has been presented in the result section. SmartPLS 3 (smartPLS, RRID: SCR_022040) was used to test the proposed hypotheses and perform the mediation analysis. Partial least squares-structural equation modeling (PLS-SEM) was used to test linear and additive models. PLS-SEM is becoming more popular in healthcare research, as it is a suitable and reliable tool for analyzing composite models in empirical studies. ^90^ In the following sections, the results are presented in the form of figures and tables.

### Ethical considerations

The ethical approval (IEC: 868/2020) was granted by “The Institutional Ethics Committee (IEC) of Kasturba Medical College and Kasturba Hospital, Manipal”. The data were kept completely undisclosed and no participant identifiers are used.

### Pilot testing

Finally, a pretest of the questionnaire was conducted on a sample of 47 outpatients in the Udupi area before the actual survey. There were no reported misconceptions or difficulties with the questions. The constructs had Cronbach’s alpha values of >0.7, specifying good levels of internal consistency such as BI (0.891), trust (0.875), PS (0.862), and PSQ (0.756). A main components analysis of the data was used to get a preliminary indication of construct validity. All factor loadings and communalities were considerably over 0.50, and “Kaiser–Meyer–Olkin and Bartlett’s test” was significant and >0.80.

## Results

The frequencies and percentages of the demographic variables were estimated using the software SPSS version 27 and the output is presented in Tables 1 & 2. The complete dataset is found in the *Underlying data.* ^85^

**Table 1.** Demographic characteristics (N=242).

Demographics	Components	N	%
**Gender**	Male	133	55.0
	Female	109	45.0
**Age**	18—25	38	15.7
	26—40	132	54.5
	41—55	54	22.3
	56—65	18	7.4
**Education**	Up to 12th	95	39.3
	Graduate	103	42.6
	Postgraduate	44	18.2
**Occupation**	Unemployed	93	38.4
	Employed	75	31.0
	Professional	47	19.4
	Business	27	11.2
**Monthly Income**	25000 and below	162	66.9
	25001-75000	64	26.4
	75001-125000	9	3.7
	125001-200000	5	2.1
	Above 200000	2	.8

**Table 2.** Outpatient Departments involved in the study.

Category	Departments	N	%
Medicine	Medicine	69	28.5
Medical Specialty	Cardiology	14	5.8
	Dental	4	1.7
	Dermatology	12	5.0
	Endocrinology	9	3.7
	ENT	14	5.8
	Gastroenterology	2	.8
	Nephrology	17	7.0
	Neurology	13	5.4
	OBG	24	9.9
	Oncology	14	5.8
	Ophthalmology	9	3.7
	Ortho	16	6.6
	Pulmonology	2	.8
	Urology	15	6.2
Surgery	Surgery	7	2.9
Surgical specialty	Neurosurgery	1	.4

### Structural model

**Figure 2.**
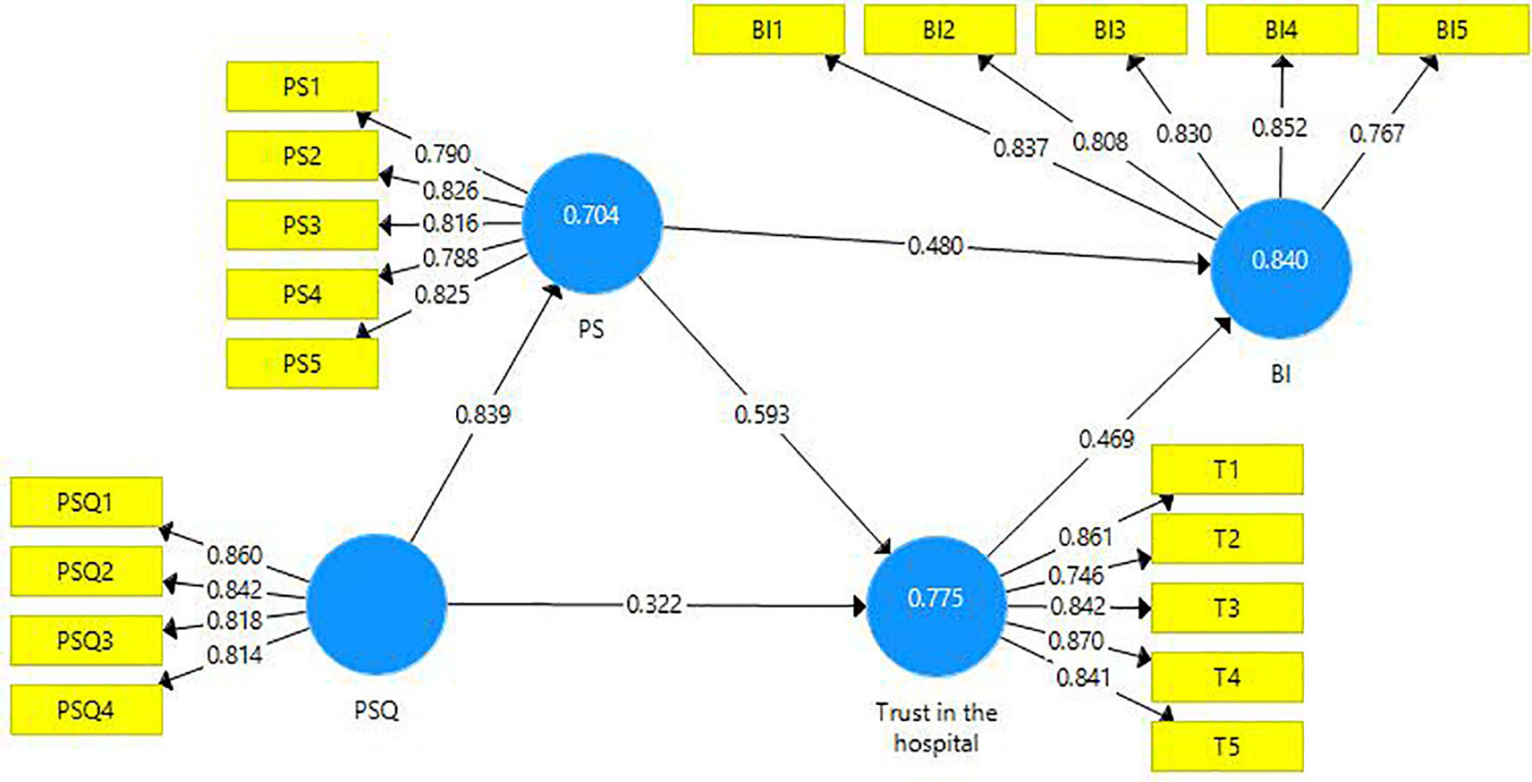
Structural model evaluation.

The structural model evaluation is represented below ( Figure 2).

The reliability and validity of constructs were investigated using outer loadings, composite reliability, average variance extracted (AVE), and variance inflated factor (VIF). ^89^ The output is presented in the table below ( Table 3). Composite reliability was used to examine the internal consistency reliability of constructs. ^91^ The composite reliability of all the constructs is above the threshold value. In PLS, the indicators were placed in order based on their reliability. Cronbach’s alpha implies that all indications are equally reliable. Cronbach’s alpha value of all the constructs was above 0.7. ^92^ AVE numbers were used to quantify convergent validity, which is the ability of a latent concept to elucidate a large portion of the variance in its indicators. The constructs’ AVEs were well above the threshold value of 0.5. ^93^

**Table 3.** Evaluation of the structural model.

Construct	Indicators	Outer loadings	Composite Reliability	AVE	Cronbach’s Alpha	Outer weights	VIF
Perceived Service Quality	PSQ1	0.860 ***	0.901	0.695	0.854	0.303 ***	2.194
	PSQ2	0.842 ***				0.317 ***	1.961
	PSQ3	0.818 ***				0.295 ***	1.837
	PSQ4	0.814 ***				0.284 ***	1.843
Patient Satisfaction	PS1	0.790 ***	0.905	0.655	0.868	0.225 ***	2.025
	PS2	0.826 ***				0.245 ***	2.107
	PS3	0.816 ***				0.269 ***	2.041
	PS4	0.788 ***				0.232 ***	1.915
	PS5	0.825 ***				0.264 ***	2.140
Trust	T1	0.861 ***	0.919	0.694	0.889	0.252 ***	2.620
	T2	0.746 ***				0.233 ***	1.635
	T3	0.842 ***				0.247 ***	2.226
	T4	0.870 ***				0.235 ***	2.808
	T5	0.841 ***				0.234 ***	2.375
Behavioral Intentions	BI1	0.837 ***	0.911	0.672	0.877	0.255 ***	2.197
	BI2	0.808 ***				0.236 ***	1.984
	BI3	0.830 ***				0.246 ***	2.177
	BI4	0.852 ***				0.250 ***	2.343
	BI5	0.767 ***				0.232 ***	1.728

*** p<0.01,

** <0.05,

* p<0.1.

### Assessment of collinearity

The guiding principle of VIF is considered to test the collinearity among the constructs. ^89^ All the constructs exhibited VIF lesser than 5. This explains that there is no problem with collinearity in the structural model. Table 4, given below, demonstrates the VIF values that this study obtained while we tested the collinearity among independent variables against their respective endogenous constructs. The VIF values of this study were obtained when the collinearity was tested among all the constructs of the inner model of this study. The process of data analysis began with the objective to find out the probability of the presence of collinearity among exogenous constructs. No collinearity was found among the exogenous constructs of this study.

**Table 4.** Evaluating collinearity for exogenous constructs through VIF inner model.

Constructs	BI	PS	Trust
**PS**	3.917	NA	3.375
**PSQ**	NA	1.000	3.375
**Trust**	3.917	NA	NA

Table 5 given below shows the VIF values of this study obtained when the collinearity was tested among all the indicators of the outer model of this study.

**Table 5.** Evaluating collinearity for indicators through VIF outer model.

Indicators	VIF	Indicators	VIF	Indicators	VIF	Indicators	VIF
**BI1**	2.197	**PS1**	2.025	**PSQ1**	2.194	**T1**	2.620
**BI2**	1.984	**PS2**	2.107	**PSQ2**	1.961	**T2**	1.635
**BI3**	2.177	**PS3**	2.041	**PSQ3**	1.837	**T3**	2.226
**BI4**	2.343	**PS4**	1.915	**PSQ4**	1.843	**T4**	2.808
**BI5**	1.728	**PS5**	2.140	**NA**	-------	**T5**	2.375

As the above tables demonstrate, there are no collinearity issues in the structural model of this research endeavor. Thus, there is no multicollinearity among either the constructs of the study (inner model) or indicators of the study (outer model). Multicollinearity is not an issue by the criterion VIF < 5. ^89^

### Discriminant validity among latent constructs: Cross-loading method

Cross-loadings (CL) of indicators constitute an additional method of assessing discriminant validity (DV). The outer loadings of indicators are expected to be greater than their CL on other constructs. ^89^ The below-mentioned table ( Table 6) demonstrates the presence of DV in the constructs of the study.

**Table 6.** Discriminant validity by the cross-loading method.

	BI	PS	PSQ	Trust
**BI1**	**0.837**	0.759	0.649	0.756
**BI2**	**0.808**	0.698	0.608	0.702
**BI3**	**0.830**	0.750	0.682	0.713
**BI4**	**0.852**	0.746	0.670	0.742
**BI5**	**0.767**	0.672	0.649	0.707
**PS1**	0.653	**0.790**	0.625	0.620
**PS2**	0.748	**0.826**	0.641	0.681
**PS3**	0.748	**0.816**	0.801	0.728
**PS4**	0.658	**0.788**	0.605	0.691
**PS5**	0.764	**0.825**	0.704	0.760
**PSQ1**	0.663	0.688	**0.860**	0.710
**PSQ2**	0.711	0.728	**0.842**	0.731
**PSQ3**	0.614	0.716	**0.818**	0.643
**PSQ4**	0.662	0.664	**0.814**	0.644
**T1**	0.790	0.739	0.693	**0.861**
**T2**	0.691	0.735	0.647	**0.746**
**T3**	0.751	0.732	0.726	**0.842**
**T4**	0.738	0.684	0.642	**0.870**
**T5**	0.704	0.700	0.699	**0.841**

### Hypotheses testing

All exogenous latent variables’ effect sizes, f ^2^, were estimated ( Table 7). The magnitude of the variables’ influence is reflected by f ^2^, regardless of the sample size. ^94^ A strong effect is reported when the effect size exceeds 0.35; a moderate effect is given when the effect size is between 0.15 and 0.35, and a small influence is recorded when the effect size is less than 0.15 stated as a low effect. PSQ on PS (f ^2^=2.375), PS on trust (f ^2^=0.464), trust on BI (f ^2^=0.352), and PS on BI (f ^2^=0.368) are assessed to have substantial effect sizes in this study. The value of the standard root mean square residual (SRMR) is used to measure model fitness. The threshold value of SRMR is <0.8. ^95^ This model’s SRMR rating is 0.066, indicating that it fits the data well.

**Table 7.** Hypothesis testing (f ^2^) and Predictive relevance test (q ^2^).

Relationship	Path Coefficient	t-Value	Bias Corrected 95% Confidence Interval	f ^2^	q ^2^
PSQ -> PS	0.839 ***	34.906	(0.781 0.878)	2.375	Not Defined
PS -> Trust	0.593 ***	7.458	(0.433, 0.748)	0.464	0.451
PSQ -> Trust	0.322 ***	3.703	(0.153, 0.493)	0.137	Not Defined
Trust -> BI	0.469 ***	6.480	(0.330, 0.493)	0.352	0.528
PS -> BI	0.480 ***	6.655	(0.333, 0.617)	0.368	0.556

*** p<0.01,

** <0.05,

* p<0.1.

### Predictive relevance test (Q ^2^)

Blindfolding is a technique for reusing samples. It allows you to calculate the Q ^2^ value, ^96^^,^^97^ which is an evaluation criterion for the PLS path model’s cross-validated predictive significance. Q ^2^ values greater than zero suggest that your data is well rebuilt and the model is predictive. The Q ^2^ values of the dependent and mediating variables are presented in the table below ( Table 7).

### Mediation analysis

**Figure 3.**
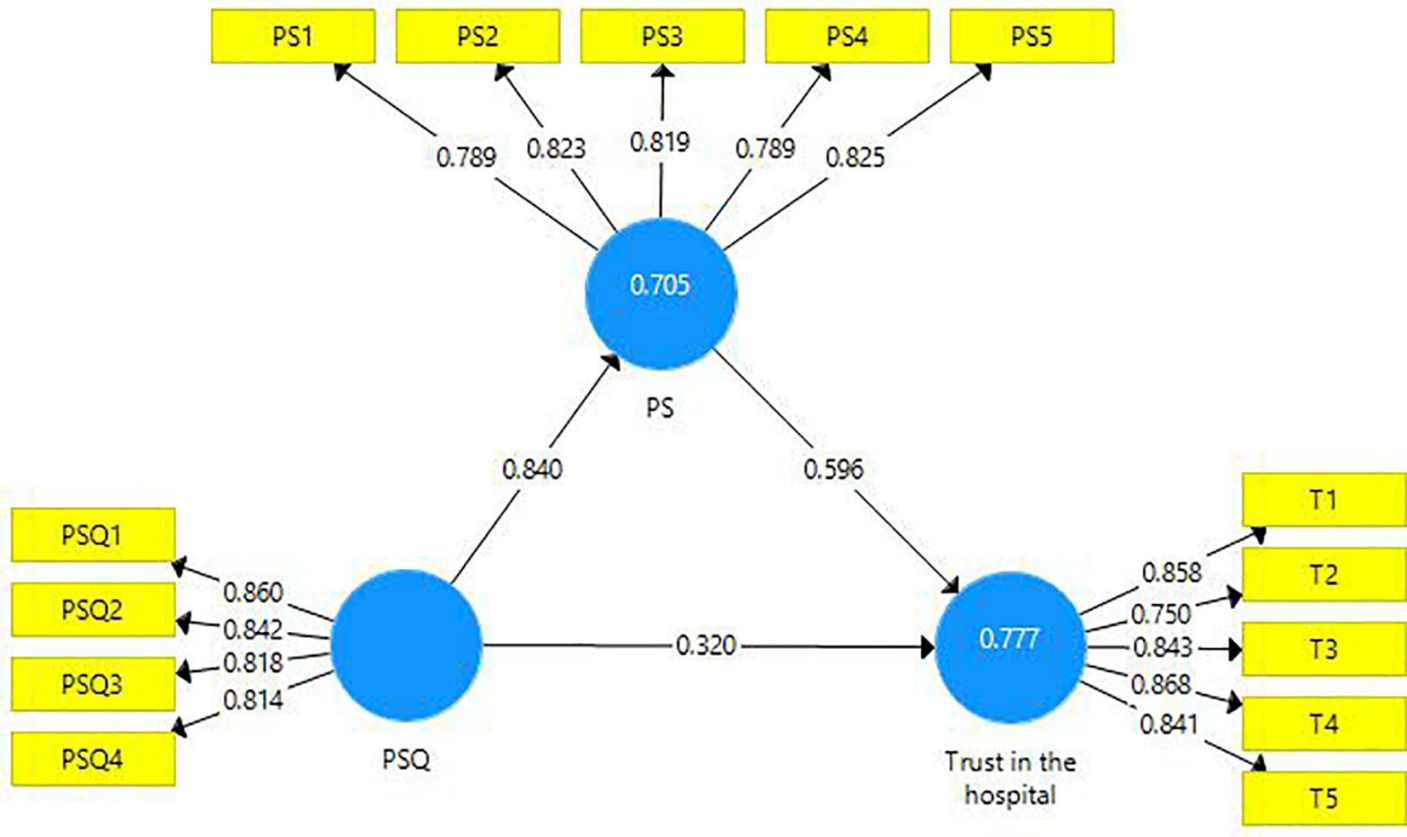
The mediating role of patient satisfaction.

**Figure 4.**
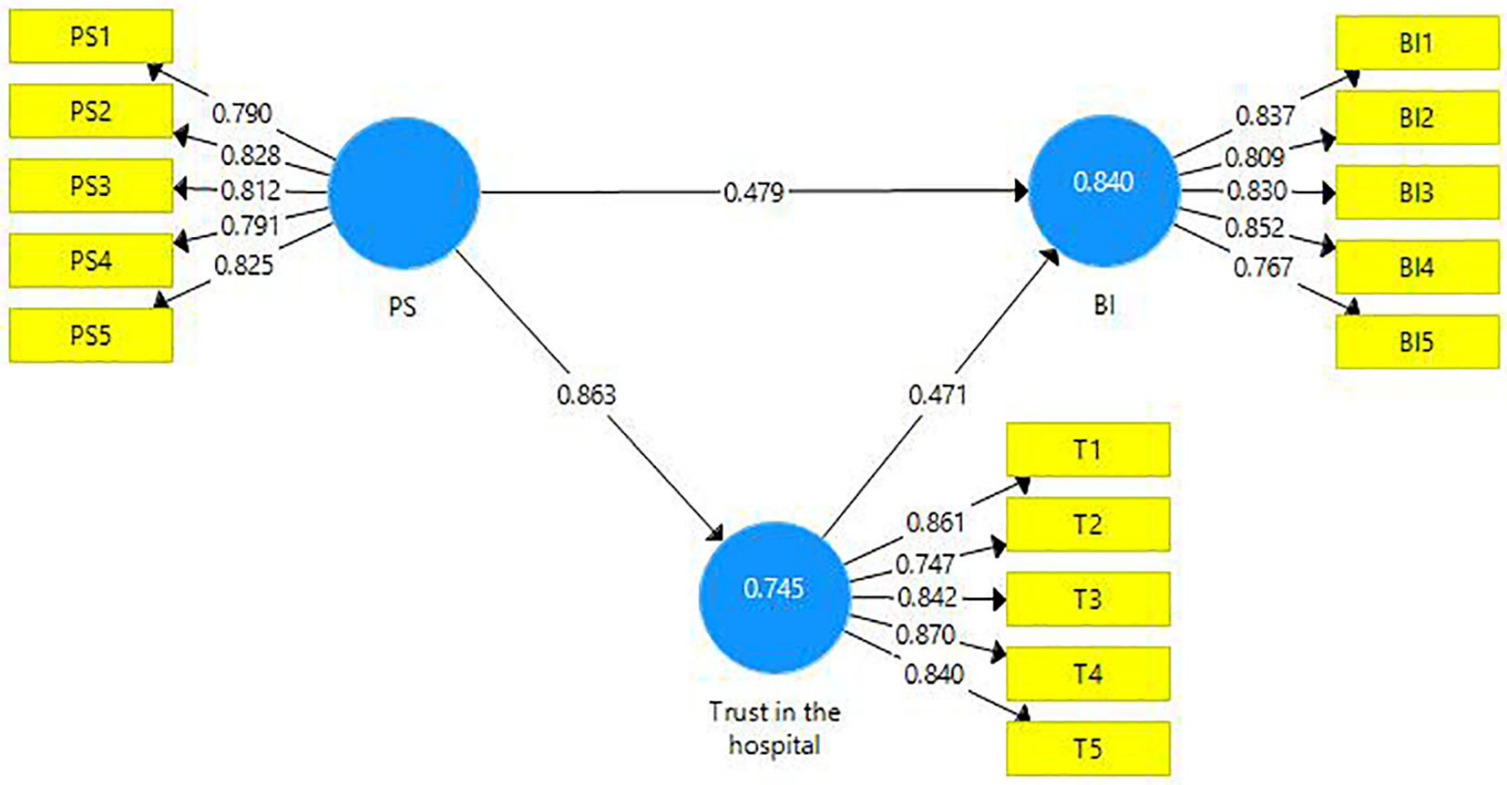
The mediating role of trust in the hospital.

This research endeavor includes two mediations. First, the latent variable PS is a mediator amongst the latent variables PSQ and trust in the hospital ( Figure 3). A partial mediation has been observed and it is presented in the table below ( Table 8). Second, the latent variable ‘trust in hospital’ is a mediating variable between the latent variables PS and BI ( Figure 4). A partial mediation has been observed and it is presented below ( Table 9).

**Table 8.** The mediating role of patient satisfaction.

	Direct Effect	Indirect Effect	VAF	Mediation
Figure 3	0.320 ***	0.501 ***	61%	Partial

*** p<0.01,

** <0.05,

* p<0.1.

**Table 9.** The mediating role of trust.

	Direct Effect	Indirect Effect	VAF	Mediation
Figure 4	0.479 ***	0.406 ***	46%	Partial

*** p<0.01,

** <0.05,

* p<0.1.

### Importance performance matrix analysis (IPMA)

IPMA allows investigators to look at an item’s importance as well as its performance. The goal of this analysis is to determine the overall influence of the preceding constructs (PSQ, PS, and trust) in predicting the endogenous construct (BI). ^89^ The total effect determines the construct’s relevance, while the performance is determined by the mean value of their score (from 0 to 100). ^98^

**Figure 5.**
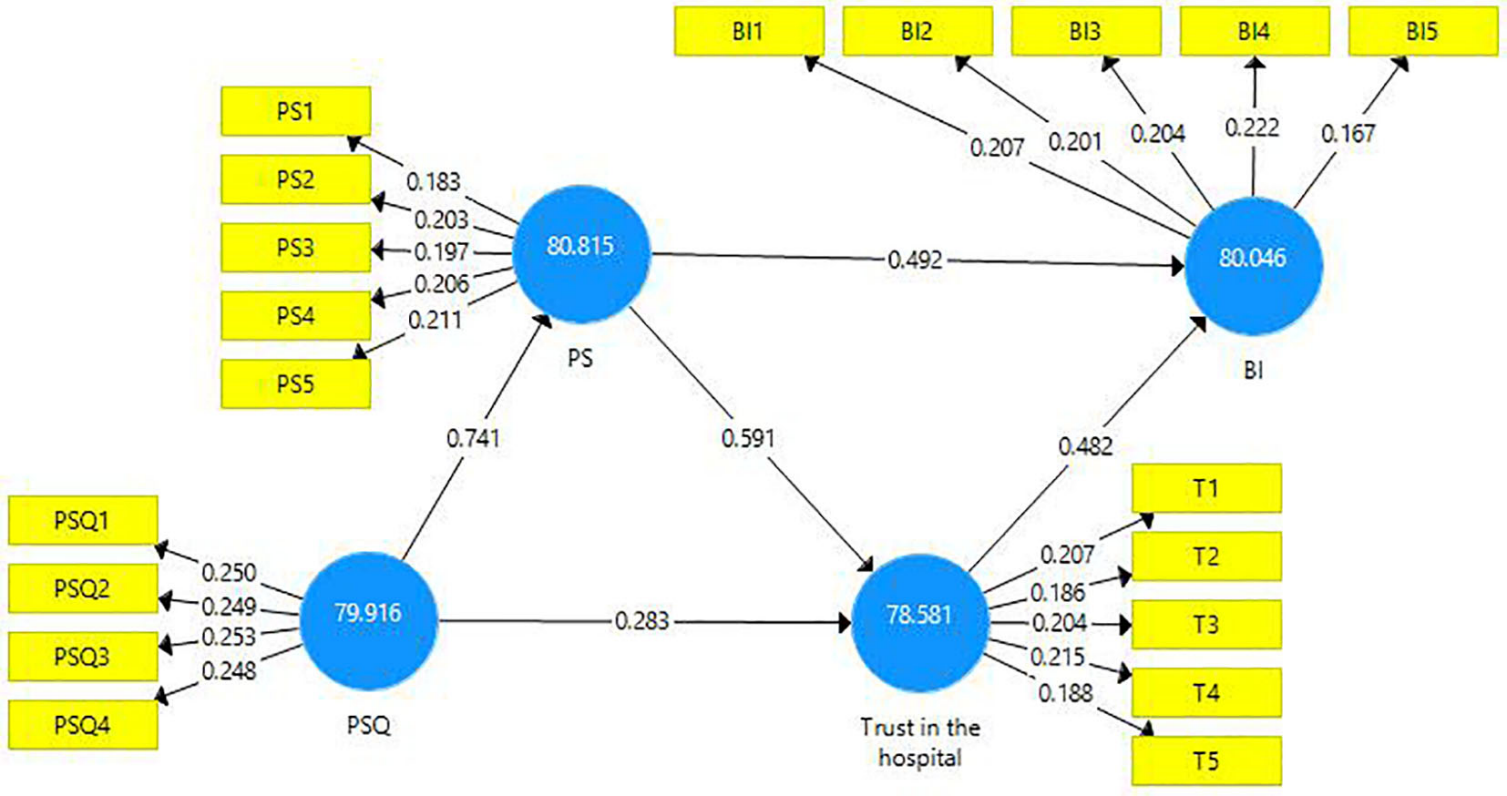
Importance performance matrix analysis.

IPMA’s findings are shown in Figure 5 and Table 10. PS outperforms the other exogenous constructs in terms of performance (80.815), according to analyses. Furthermore, PS has a total effect on BI of 0.759, which is quite strong. As a result, a unit increase in PS performance from 80.815 to 81.815 results in a performance gain in BI from 80.046 to 80.805. The exogenous construct PSQ has a total effect of 0.787 and a performance of 79.916. As a result, a one-unit improvement in PSQ from 79.916 to 80.916 would boost BI’s performance from 80.046 to 80.833. Similarly, the exogenous construct trust has a total effect of 0.469 and a performance of 78.581. As a result, a one-unit increase in trust from 78.581 to 79.581 would result in an increase in BI to 80.515. PS has a robust and significant influence on BI, followed by PSQ and trust. This has significant implications for healthcare practitioners.

**Table 10.** IPMA of Institutional Efficacy.

	Behavioral Intention (BI)	
Latent Constructs	Importance (Total Effects)	Performance (Index Values)
Patient satisfaction (PS)	0.759	80.815
Perceived service quality (PSQ)	0.787	79.916
Trust	0.469	78.581

### Importance performance map

**Figure 6.**
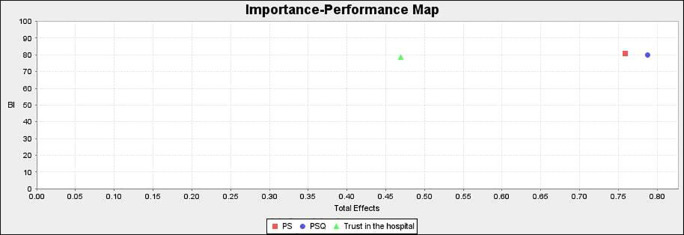
Importance performance map.

Figure 6 shows the performance of the exogenous constructs. PS is high on performance whereas trust in the hospital is low on performance.

## Discussion

In this research endeavor, the researchers observed that PS and trust in the hospital play a vital role in developing BI. In adverse services, PSQ and PS support building trust in the hospital. When a patient trusts the hospital, he/she intends to visit the hospital again for their treatment. PSQ, PS, and trust in the hospital are explored as an antecedent to BI. This study concludes that patient who is satisfied with the healthcare services received will develop trust in that hospital and continues to visit the hospital for treatment in the future.

In this study, a partial mediation relationship is observed between PSQ and trust in the hospital. Also, there is a partial mediating relationship between PS and BI. This study consists of a conceptual framework; the hypotheses were proposed based on the literature review explaining the association between the constructs. The proposed model is empirically tested, and validity and reliability of the constructs were estimated. Hypotheses testing and mediation analysis were done. IPMA was done to estimate the performance of endogenous variables through the exogenous variables.

The objective of this paper is to investigate the influence of PSQ on BI through PS and trust in the hospital. This paper includes a conceptual framework and hypotheses. The first hypothesis was that PSQ and PS are significantly related to each other. This hypothesis is in line with the literature that shows a significant relationship between healthcare quality and PS. ^43^ Secondly, researchers hypothesized that PS is positively related to trust. It was shown by researchers in the past that PS is a multi-dimensional element of the healthcare sector that influences patient trust. ^12^^,^^43^ Past literature has shown that satisfaction is an antecedent of trust. ^70^ Further, it was hypothesized that the association between PSQ and trust in the hospital is mediated by PS. PS results from healthcare SQ which in turn leads to trust. PS is a processing feature of trust. ^12^ In the service sector, the influence of SQ on building trust will be through consumers’ satisfaction. ^72^

The next hypothesis states that trust in the hospital is significantly associated with BI. Past studies have explored the relationship between trust and BI in the marketing domain. ^26^^,^^30^^,^^49^^,^^73^ Past empirical research recommended that trust is essential to establish BI, especially in service sectors. ^46^^,^^75^^,^^76^ Scholars have suggested that building trust in any institution results in repeat business. ^29^ Last, the researchers hypothesized that trust in the hospital acts as a mediator between PS and BI. Scholars have observed that trust plays a mediating role in service entities. ^50^^,^^77^^,^^78^ Trust is exhibited as a mediator between perceived quality and intent to visit for medical treatment. ^16^ IPMA determines that the performance of the construct PS is higher than the other exogenous constructs concerning BI. Trust in the hospital is low on performance. Therefore, it is important to build trust in the hospital and enhance behavioral intent.

The findings of the study imply a few managerial implications that are mentioned here. This study suggests that service providers should obtain customer input on the services received. Service providers should deliver error-free services to ensure satisfaction. It also aids in matching and comprehending customer expectations. The staff’s readiness to serve the patients is demonstrated by their willingness to provide service without being asked. Measurement of healthcare service quality should include factors such as personnel technical experience, availability of additional amenities such as medical equipment, and availability of various types of drugs. Hospitals should give effective training to all employees including nurses, doctors, and general staff to improve their communication so that they can deliver excellent service to patients. In a hospital, it is important to deliver the services to the best, which improves PS. Medical professionals should be consumer-oriented and deliver high-quality services with a human touch. Along with the quality of services, maintaining a patient-provider relationship is also very essential. This relationship becomes the base of developing trust in the hospital. This trust then influences repeat visits which aid in profitability. Therefore, trust in the hospital plays a vital role in enhancing the BI of a patient.

## Conclusion

This study provides several insights for healthcare organizations to develop BI by enhancing trust in the hospital. A hospital can assure the satisfaction of patients when good quality services are rendered by healthcare professionals. Satisfied patients develop trust in the hospital and this becomes the influencing factor of revisit intention. PS has a significant mediating effect between perceived service quality and trust in the hospital. Trust in the hospital has a significant mediating effect between PS and BI. Thus, we can conclude that improving PS and trust in the hospital is essential to having a positive intention to revisit the healthcare organization.

Unlike other studies, this study also has a few limitations which pave the way for future researchers. First, this study was conducted in outpatient departments of multispecialty hospitals. Future researchers would do a comparative study including both inpatients and outpatients. Second, the hospitals were situated in higher HDI districts. Future researchers would consider lower as well as higher HDI districts and carry out a comparative study.

## Data Availability

Figshare: Underlying data: A Cross-sectional study on Exploring the Antecedents of Patient’s Revisit Intention: Mediating role of Trust in the Hospital among Patients in India https://doi.org/10.6084/m9.figshare.21505641. ^85^ This project contains the following underlying data:
• Dataset.csv This project contains the following extended data:
• Questionnaire.docx
