# Supplementary material for: A cross-sectional study on exploring the antecedents of patient’s revisit intention: Mediating role of trust in the hospital among patients in India
Source: F1000Res. 2024 Mar 18;12:75. Originally published 2023 Jan 19. [Version 3] doi: 10.12688/f1000research.128220.3 (PMC10928416; doi:10.12688/f1000research.128220.3)
Supplement: Peer review history

# Reviewer report: Reviewer response for version 2

**DOI:** 10.5256/f1000research.153946.r239867

**Published:** 2024-03-11

**Authors:** Satyanarayana Rentala

**Affiliations:** 1 Bharathidasan Institute of Management, Tiruchirappalli, Tamil Nadu, India

The revised manuscript addresses all the issues highlighted by the earlier reviewers. The manuscript may now be considered. The research design and methodology are adequate. The results of the research model have been explained well. The literature review covers the important, and recent research studies. The data set had been provided by the authors to check for replicability and other analytical aspects of the research.

Is the work clearly and accurately presented and does it cite the current literature?

Yes

If applicable, is the statistical analysis and its interpretation appropriate?

Yes

Are all the source data underlying the results available to ensure full reproducibility?

Yes

Is the study design appropriate and is the work technically sound?

Yes

Are the conclusions drawn adequately supported by the results?

Yes

Are sufficient details of methods and analysis provided to allow replication by others?

Yes

Reviewer Expertise:

Export performance of Indian pharmaceutical industry; Service quality in Indian healthcare (hospital industry); Financial inclusion; Sustainable Development Goals; Sustainable and Responsible Marketing

I confirm that I have read this submission and believe that I have an appropriate level of expertise to confirm that it is of an acceptable scientific standard.

# Reviewer report: Reviewer response for version 2

**DOI:** 10.5256/f1000research.153946.r195437

**Published:** 2023-08-31

**Authors:** Prof. (Adj.) Dr. Supaprawat Siripipatthanakul

**Affiliations:** 1 Bangkokthonburi University, Bangkok, Bangkok, Thailand; 2 Manipal GlobalNxt University, Nilai, Negeri Sembilan, Malaysia

Summary of revisions:

 The following changes are incorporated in the manuscript: 

 1. reference quoted for sample size determination

 Reviewer's Feedback: Acceptable 

 2. mentioned limitations as directed by reviewer  

 Reviewer's Feedback: Acceptable 

 3. strengthened the implications in the discussion section

 Reviewer's Feedback: Acceptable

Is the work clearly and accurately presented and does it cite the current literature?

Partly

If applicable, is the statistical analysis and its interpretation appropriate?

Yes

Are all the source data underlying the results available to ensure full reproducibility?

Partly

Is the study design appropriate and is the work technically sound?

Partly

Are the conclusions drawn adequately supported by the results?

Partly

Are sufficient details of methods and analysis provided to allow replication by others?

Partly

Reviewer Expertise:

Management (Business, Education, Healthcare) & Behavioural Analytics

I confirm that I have read this submission and believe that I have an appropriate level of expertise to confirm that it is of an acceptable scientific standard.

# Reviewer report: Reviewer response for version 1

**DOI:** 10.5256/f1000research.140789.r176087

**Published:** 2023-07-20

**Authors:** Prof. (Adj.) Dr. Supaprawat Siripipatthanakul

**Affiliations:** 1 Bangkokthonburi University, Bangkok, Bangkok, Thailand; 2 Manipal GlobalNxt University, Nilai, Negeri Sembilan, Malaysia

The topic should include patient satisfaction as another mediator. Please consider adding new citations and references because all previous studies are from 1974–1998 and 2006–2019. There are no recent studies for the years 2020–2023. It is hard to say that sufficient details of methods are provided due to the research design, which is necessary to be more straightforward for sample size determination. It is better to explain a definite or indefinite population of hospital outpatients. The data is from three multispecialty hospitals in Bangalore Urban and one in Mysore from August 4th, 2021, to August 26th, 2021. It means that it is a definite population, and it is necessary to calculate it from a known population of three hospitals. However, the sample size was 242; still, previous studies must support this number. In this case, the researchers did not use sample size determination calculations from a definite population. The statistical analysis and its interpretation are acceptable. The conclusion should summarize how a strategic planner could improve each variable, especially perceived service quality, because the researchers do not show the questions. The limitations could not be explained in the present because the data was collected in 2021, but now it is 2023 as current, and the data was explained in 2021, not now. It is necessary to mention this in the limitations section.

Is the work clearly and accurately presented and does it cite the current literature?

Partly

If applicable, is the statistical analysis and its interpretation appropriate?

Yes

Are all the source data underlying the results available to ensure full reproducibility?

Partly

Is the study design appropriate and is the work technically sound?

Partly

Are the conclusions drawn adequately supported by the results?

Partly

Are sufficient details of methods and analysis provided to allow replication by others?

Partly

Reviewer Expertise:

Management (Business, Education, Healthcare) & Behavioural Analytics

I confirm that I have read this submission and believe that I have an appropriate level of expertise to confirm that it is of an acceptable scientific standard, however I have significant reservations, as outlined above.

# Response

**Published:** 2023-07-31

**Authors:** Nahima Akthar

**Affiliations:** Manipal Academy of Higher Education, India

Dear Sir,

 Many thanks for the review. We are hereby enclosing our response to your queries.

- Patient Satisfaction as a mediator: The mediating role of “patient satisfaction” as a construct is explored and presented in the manuscript. PLS SEM software is used to assess the mediating effect and the output is presented in Figure 3 and Table 8.
- References: This manuscript was conceptualized, and data collection was completed in 2021. Data cleaning, analysis, and drafting of the manuscript were undertaken in 2022 and the final version was submitted to the journal on November 5 ^th,^ 2022. Hence, we have relied on the published literature until 2021.
- Sample size determination: we have incorporated this in our manuscript. The reference was not presented, which had been incorporated now. The explanation for the same is presented below:
  PLS-SEM is the software adopted to undertake the analysis. This investigation used a purposive sampling approach (non-probability by nature), which was adopted due to the absence of a sampling frame. Data was collected from 231 respondents. When the PLS-SEM is used for data analysis, Avkiran (2018) suggests that the sample size is determined by multiplying the number of items on the rating scale by 10. Hence 21 ( number of items) * 10 = 210. In addition 10% error of margin therefore 231.
  Ref: Avkiran NK: An in-depth discussion and illustration of partial least squares structural equation modeling in health care. *Health care management science.* 2018 Sep;21(3):401–408. 28181112 10.1007/s10729-017-9393-7
- Implications: emphasized in the manuscript
- Limitation: change incorporated in the manuscript

Regards,

 Authors
